# 4D Printing Strain Self‐Sensing and Temperature Self‐Sensing Integrated Sensor–Actuator with Bioinspired Gradient Gaps

**DOI:** 10.1002/advs.202000584

**Published:** 2020-05-13

**Authors:** Daobing Chen, Qingping Liu, Zhiwu Han, Junqiu Zhang, HongLie Song, Kejun Wang, Zhengyi Song, Shifeng Wen, Yan Zhou, Chunze Yan, Yusheng Shi

**Affiliations:** ^1^ State Key Laboratory of Material Processing and Die & Mould Technology Huazhong University of Science and Technology Wuhan Hubei 430074 China; ^2^ Key Laboratory of Bionic Engineering Ministry of Education Jilin University Jilin Changchun 130022 P. R. China; ^3^ Applied Mechanics Laboratory Department of Engineering Mechanics Tsinghua University Beijing 100083 China; ^4^ Faculty of Engineering China University of Geosciences Wuhan Hubei 430074 China

**Keywords:** 4D printing, bioinspired gradient gaps, integrated sensor–actuators, strain self‐sensing, temperature self‐sensing

## Abstract

Integrated sensor–actuators with exciting functionalities, such as action self‐sensing, position self‐sensing, posture self‐sensing, or active sensing, are promising for applications in biomedical device, human–machine interaction, intelligent self‐protection devices, and humanoid robots. Despite recent progress, it remains challenging to achieve a macroscopical integrated sensor–actuator in a material system with microstructures. To address this critical challenge, a 4D printing bioinspired microstructure strategy is reported to design a high‐performance integrated sensor–actuator capable of simultaneous actuation and sensation. Decoupled thermal stimulation and strain sensation is achieved by combining nanocarbon black/polylactic acid composites with bioinspired gradient microgap structures. As a result, printed integrated sensor–actuators can actively touch objects triggered by thermal stimulation and self‐sense the touching state through the resistance change. It is anticipated that the basic design principle underlying this behavior can be used to develop integrated sensor–actuators of various shapes and functionalities to meet desirable applications.

## Graphical abstract

A printable integrated sensor–actuator (PISA) with bioinspired gradient gaps is reported. Using 4D printing, the bionic structure is creatively combined with smart materials to obtain the sensor–actuator components with self‐temperature sensing and active touching strain feedback. This PISA and its design principle provide a promising approach to fabricate smart devices in future humanoid system.

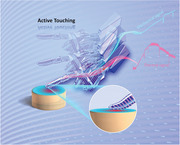

Artificial intelligent devices are commonly produced by assembling components. In contract to synthetic systems, biomaterials often combine multifunction into one block by integrating at material level.^[^ ^1^, ^2^ ^]^ In the integrated devices, materials and structures play similar roles as components and mechanisms in traditionally assembled devices. The integrated multifunction materials have several remarkable advantages such as lightweight, voluminal efficiency, and robustness, which are often more time‐effective and cost‐effective during the manufacturing process.^[^ ^3^, ^4^ ^]^ However, it is difficult to produce these integrated materials with intricate internal structures and multifunction.^[^ ^5^ ^]^ With the emerging of 3D and 4D printing technologies, they provide an exciting promise approach to fabricating these biomimetic multifunctional materials.^[^ ^6^ ^]^ Particularly, 4D printing technology combines stimuli‐response materials with 3D printing process, which provides an unprecedented way to create programmed smart materials in one step. With the above in mind, we conceive of developing a monolithic sensor–actuator by 4D printing technology.

In recent years, independent sensors or actuators have been widely studied and various breakthroughs have been made.^[^ ^7^, ^8^, ^9^, ^10^, ^11^ ^]^ Especially, the emerging of sensor functional materials with micro/nanostructures, such as serpentine structure,^[^ ^12^ ^]^ bulking,^[^ ^13^ ^]^ wrinkle,^[^ ^14^ ^]^ interlock,^[^ ^15^ ^]^ pyramid,^[^ ^16^ ^]^ and bioinspired slit/crack/gap,^[^ ^17^, ^18^, ^19^ ^]^ with different material processing methods, such as self‐assembly, lithography, mechanical processing, and stress inflation,^[^ ^12^, ^13^, ^14^, ^15^, ^16^, ^17^, ^18^, ^19^ ^]^ has considerably improved the sensitivity, response speed, reliability, and resolution of various sensors. Meanwhile, the emerging of actuator functional materials, such as conducting polymers,^[^ ^20^, ^21^ ^]^ multilayer paper composite materials,^[^ ^3^ ^]^ ionic soft actuators,^[^ ^4^, ^22^ ^]^ dielectric elastomers,^[^ ^23^, ^24^, ^25^ ^]^ liquid‐crystal elastomers,^[^ ^26^, ^27^ ^]^ liquid‐metal composite materials,^[^ ^28^, ^29^ ^]^ and magnetic composite materials,^[^ ^30^ ^]^ has considerably improved the deformation amplitude, response speed, force generation, and programmable motion output of various actuators. Aside from high‐performance sensors and efficient shape‐changing behavior of actuators, an integrated sensor–actuator with exciting properties such as action self‐sensing, position self‐sensing, posture self‐sensing, or active sensing is essential for their greater functionalism and wider adoption. However, the vast majority of above actuators and sensors are independent of each other at present; that is, when actuators perform their actions, they cannot self‐perceive their position, posture, and environmental change (Table S1, Supporting Information).

Most recently, a few studies have demonstrated actuators with integrated mechanical‐sensing elements.^[^ ^3^, ^4^, ^31^, ^32^, ^33^ ^]^ The strategy of latest work of the integrated sensor–actuators has adopted the layered structure of the thin films, in which the sensing material and the actuator material were laminated together.^[^ ^3^, ^4^ ^]^ For example, Amjadi and Stti coated graphite‐carbon nanotube on paper and integrated it with polypropylene (PP) material to form a two‐layer structure film, and have made use of the different expansion rates of the two‐layer structure film and the sensing properties of graphite‐carbon nanotube to prepare self‐sensing executive function structural materials.^[^ ^3^ ^]^ Phan et al. have reported a flexible actuator based on paper coated with ferromagnetic nanoparticles and graphite.^[^ ^31^ ^]^ In another work, graphene mesh has been embedded in the ionic soft actuator forming a sandwich structure for strain sensing in a self‐sensing actuator.^[^ ^4^ ^]^ This laminated strategy can realize the sensor–actuators to a certain extent, but there are still many problems, such as poor interface integration and difficulty in complex shape manufacturing. Therefore, in order to obtain an ideal integrated sensor–actuator, micro/nanostructures should be macroscopically manufactured with a material system.

Herein, we presented a printed integrated sensor–actuator (PISA) with bifunctional sensing and self‐sensing actuating in a material system. The PISA was fabricated by a fused deposition modeling (FDM) printer with a formulated composite material based on carbon black (CB) and polylactic acid (PLA). The gradient bioinspired gap structure from the bottom to the top in the PISA was achieved by 4D printing strategy. The PISA showed good sensitivity and stability as temperature and mechanical sensors, respectively. The gauge factor (GF) reached 48.46, which was comparable with previous self‐sensing actuators. The temperature sensing sensitivity was about (1.90–2.25) × 10^4^ ppm °C^−1^. The resistance change of the PISA was successfully explained by the electronic tunneling effect theory. When stimulated by coupling, the coupled signals could be separated and extracted. Besides, the PISA could self‐sense the temperature and shape change when performed their actions. The PISA could actively touch the mobile phone screen and convert the mechanical signal into resistance changes.

In order to print the PISA, the 4D printing materials were prepared first, the main components of which were CB nanoparticles (Figure S1, Supporting Information) and PLA (**Figure** **1**a). Carbon black composite polymer materials were regarded as a kind of excellent multifunctional intelligent material because of their unique temperature resistance and pressure resistance characteristics.^[^ ^34^, ^35^, ^36^, ^37^ ^]^ PLA was a biodegradable polymer with good shape memory that could be used to create actuators of a variety of shapes by 3D printing.^[^ ^20^, ^21^ ^]^ The main process and method of the synthesized CB/PLA composite material can be found in the Experimental Section.

**Figure 1:**
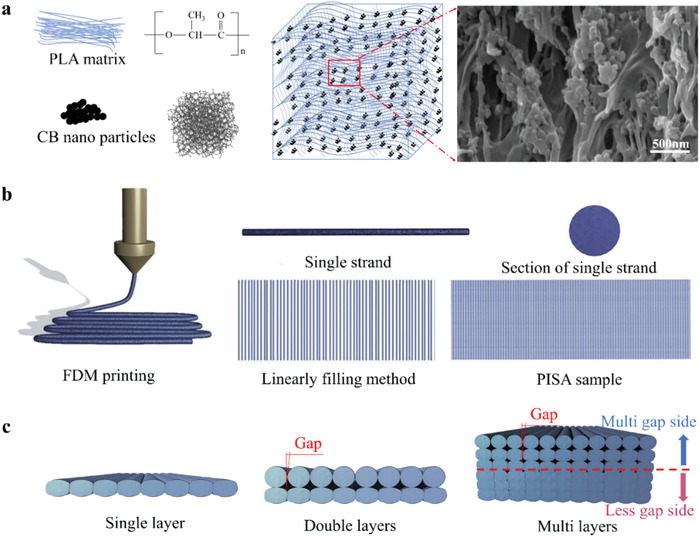
Schematic of the CB/PLA composite materials and the 4D printing methods. a) The CB nanoparticles were dispersed in PLA matrix. Many gaps were formed between CB nanoparticles because of the barrier of PLA matrix. b) The PISA sample was fabricated by an FDM with linearly filling method. c) In a PISA sample of double or multilayers, gap structures were formed between the filaments of the upper layers. The gap width had the characteristics of increasing gradient from bottom layer to top layer.

The CB nanoparticles were dispersed in the PLA matrix with many nanogaps (Figures S2 and S3, Supporting Information). The PISA was printed by FDM with the prepared CB/PLA composite material via a linearly filling method (Figure 1b and Figure S4, Supporting Information). The PISA had a good electrical conductivity and could be used as a touching pen for triggering the mobile phone screen (Movie S1, Supporting Information). By setting printing parameters, a gradient gap structure from the bottom layer to the top layer of the PISA could be produced (Figure 1c and Figures S5–S7, Supporting Information). The gap was regarded as a defect in the field of 3D printing, while it had unusual uses in the biological receptors. For example, scorpions were found to be extremely sensitive to mechanical stimuli thanks to its gap structure in lyriform organ.^[^ ^17^, ^18^, ^19^ ^]^ Scorpions sense minute mechanical stimuli with a gap‐shaped lyriform organ near their leg joints for detecting prey (Figure S8, Supporting Information). The gaps were embedded into the exoskeletons of scorpion legs, and deformed when stimulated by mechanical stimuli.^[^ ^2^, ^38^ ^]^ This provided us with a natural system as a model for artificial sensors. The gaps could be used to mimic the gap structure of the scorpion gap‐shaped lyriform organ and improve the mechanical sensitivity of the PISA.

The PISA samples were tested as a passive sensor first. When exerting a tiny mechanical stimulus, the sensor deformed and the contact areas between the strands changed. As the PISA sample was compressed from the top layer to the bottom layer, the gaps aggregated and reconnected, and the gap (the width of the gaps between the strands) became smaller. As the strand surfaces of the gaps reconnected, electrons could pass through the CBs of closely connected regions of the ellipse surface and pass by the electron tunneling effect, resulting in a drop in resistance (**Figure** **2**a). On the other hand, as the PISA sample was compressed from the bottom layer to the top layer, the gaps disconnected and became bigger, resulting in rise in resistance (Figure 2b and Figure S9, Supporting Information). This situation was similar to the deformation of the lyriform organ,^[^ ^38^ ^]^ in which the gaps of both the PISA and scorpion connected and disconnected, submitting to compress stress. In order to evaluate the sensitivity and the frequency response properties of the PISA to strains, a home‐made system containing positioning controlled motorized linear stages and a data acquisition system were designed (Figure S10, Supporting Information). The system could provide external strain, with the resistance of the sensor being recorded simultaneously. As shown in Figure 2c, the resistance of the sensor dropped under compressing strains as the pressing depth increased. For a more precise understanding of sensitivity, the normalized resistance change Δ*R*/*R*_0_ (where Δ*R* = *R*_1_ − *R*_0_, with *R*_1_ and *R*_0_ being the resistances under compressed and relaxed states) was recorded. Δ*R*/*R*_0_ decreased monotonically, while it was not linearly, but it was closely exponential decay. Moreover, the GF, a characteristic parameter of sensitivity, derived from GF = (Δ*R*/*R*_0_)/Δ*ε*, where *ε* (Supporting Theory, Supporting Information) is the mechanical strain of the PISA, was calculated. In Figure 2c, the GF between strains of 0% and 0.7% displayed a linear slope of 48.46, which was much lower than the bionic sensors,^[^ ^13^, ^14^, ^15^ ^]^ but was comparable with previous self‐sensing actuators^[^ ^3^ ^]^ (Table S1, Supporting Information). The dynamic mechanical stimuli test results showed that the PISA could relatively accurately reflect different waveform signals, especially discontinuous and low frequency signals (Figure S11, Supporting Information), such as the square wave signal. The PISA responded quickly under the maximum displacement of 8 µm with frequencies of 10, 5, and 2 Hz (Figure 2d). Also, the PISA responded quickly under the same frequency of 3 Hz with different maximum displacements of 4, 6, and 8 µm (Figure 2e). Figure 2f shows the repeatability of the pressure sensing property of the sensor after 10 000 bends and compression.

**Figure 2:**
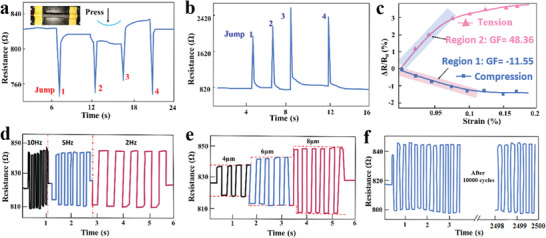
Mechanical stimulus sensing performance of the PISA. a) Pressing the PISA from the multigap side could induce the resistance decreasing jump (defined as compression mode). b) Pressing the PISA from the less gap side could induce the resistance increasing jump (defined as tension mode). c) PISA resistance responded to various compress depths when pressed from the multigap side or from the less gap side. The decreased resistance showed the exponential decay curve. The GF was about −11.55 in the decreased lineally Region 1 of the compression mode and the GF was about 48.46 in the increased lineally Region 2 of the tension mode. d) The PISA resistance responded to dynamic mechanical square pressing displacement at frequency of 10, 5, and 2 Hz. e) The PISA resistance responded to dynamic mechanical square pressing displacement at pressing displacement of 4, 6, and 8 µm. f) Stability of the PISA after 10 000 bends and compression at pressing displacement of 8 µm and frequency of 4 Hz.

Then, the temperature‐resistance characteristics of the PISA were tested. At room temperature, the PISA sample was placed on a heat table and the resistance change process was recorded simultaneously. The PISA sample was put on a heating table and heated to the preset maximum temperature (Max. *T*). As shown in **Figure** **3**a, the PISA was about 30 °C when it was just put on the heating table with the initial resistance about 1080 Ω, the resistance increased exponentially until reached the preset Max. *T*. Together, the resistance of the PISA in the cool stage was also recorded after being taken away from the heating table, and the resistance decreased exponentially until reached the room temperature (Figure 3b). In order to better understand the temperature‐resistance characteristics of the PISA, different preset Max. *T* tests were carried out. The heating table was heated to preset Max. *T* of 60, 80, 100, 120, 150, and 200 °C, respectively (Figure S12, Supporting Information). When the Max. *T* was lower (≤100 °C), the resistance–time curve increased to a certain value and kept at the value over time. When the Max. *T* was higher (≥120 °C), the resistance–time curve first increased and then decreased, and finally stabilized at a certain value. Then, the PISA sample was removed from the heating table and cooled naturally; the resistance of the PISA sample decreased exponentially over the time (Figure S12, Supporting Information). From the perspective of the resistance–temperature, as shown in Figure 3c, the resistance of the PISA sample increased with the temperature increasing within 20–120 °C and decreased with the temperature increasing within 120–200 °C. This test demonstrated the CB/PLA composite materials had positive temperature coefficient (PTC) resistance characteristics at the lower temperature and negative temperature coefficient (NTC) resistance characteristics at the higher temperature.^[^ ^34^, ^35^ ^]^ The temperature coefficient of resistivity (TCR) was usually used to indicate the temperature sensitivity of the material. TCR represented the relative change of resistance value when the temperature changed by 1 °C. The TCR value could be calculated by *ρ*_TCR_ = (*R*_1_−*R*_0_)/*R*_0_(*T*_1_−*T*_0_), where *R*_0_ is the resistance at temperature *T*_0_ and *R*_1_ is the resistance at temperature *T*_1_. As shown in Figure 3c, the *ρ*_TCR_ of the PISA in 25–120 °C was about 1.90 × 10^4^ ppm °C^−1^, and the *ρ*_TCR_ of the PISA in 120–200 °C was about 2.25 × 10^4^ ppm °C^−1^.

**Figure 3:**
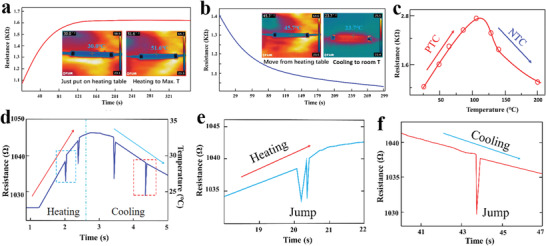
Temperature sensing performance and bifunctional sensing performance of the PISA. a) The sample resistance increased exponential decay as the temperature increased. b) The sample resistance decreased exponential decay as the temperature decreased. c) The PISA sample had positive temperature coefficient (PTC) resistance characteristics at the lower temperature (25–120 °C) and negative temperature coefficient (NTC) resistance characteristics at the higher temperature (120–200 °C). d) The PISA sample could reflect the mechanical stimulus while the temperature changed. e) As the temperature of the sample increased, the resistance increased. At the same time, the resistance jumped when the mechanical stimulus was applied. f) As the temperature of the sample decreased, the resistance decreased. At the same time, the resistance jumped when the mechanical stimulus was applied.

In order to explore the thermo‐mechanical coupling sensing characteristics of the PISA, a method with heating and touching simultaneously was adopted. The PISA sample was put on a heating table with 50 °C; the resistance of the sample increased during the heating process. Then on touching the PISA sample with a pen, the changing resistance reflected the touching information. Because it took a long time for the sample to reach thermodynamic equilibrium on the heating table, and before that, the sample was heated, the resistance increased gradually (Figure S13, Supporting Information). Specifically, the time–resistance curve in Figure 3d shows that the resistance of the sample was about 1024 Ω at 22 °C. In the process of heating, the resistance increased with temperature rising and the average increasing rate of resistance was about 15 Ω s^−1^. At the 2 s, the sample was touched for the first time, the resistance changed rapidly from 1038 Ω to 1032 Ω, and then the resistance increasing still maintained the original increasing rate (Figure 3e). The same regular curve could be obtained by moving the sample away from the heating table and touching it with a pen (Figure 3f and Figure S14, Supporting Information). The resistance jump induced by the touching could reflect the contact time and force; at the same time, the resistance of the temperature change in this short time could also be extracted.

Resistance changes of the PISA caused by mechanical stimulus or temperature stimulus essentially were induced by changing the spacing between conductive CB nanoparticles.^[^ ^17^, ^18^, ^19^, ^34^, ^35^, ^36^, ^37^ ^]^ Before being mixed, CB nanoparticles contacted with each other and formed a conductive path. After being mixed, from the cross section (Figures S2 and S3, Supporting Information) of the CB/PLA composite by scanning electron micrograph (SEM), some CB particles formed conductive links in PLA matrix, and most of them were gap distribution. When the gap between conductive particles was within 1 nm or the conductive particles were in contact with each other, the conductive path could be formed in the composite material, and the conductive polymer materials showed the conductive behavior in the macroscopic.^[^ ^36^, ^39^ ^]^ At the same time, when the gap between the conductive particles was larger, though the conductive path could not be formed, the conductive composite polymer materials still had significant conductive behavior. In this situation, the electronic tunneling effect theory could explain this phenomenon.^[^ ^36^, ^39^, ^40^ ^]^ Therefore, in our research, CB fillers were more suitable for the interpretation of tunneling effect because they had fewer conductive paths and larger gaps (the gap size was larger than 1 nm) in PLA. According to Sheng P's formula based on the theory of electronic tunneling effect, the resistivity *ρ* of the CB/PLA composite related to the time *t* could be expressed as^[^ ^39^, ^40^ ^]^ (Theory S1, Supporting Information)

(1) $$\rho \left(t\right)=\rho_{0}\;\exp\left[\frac{M\cdot \omega \left(t\right)}{T\left(t\right)+N}\right]$$

where *ρ*_0_ is the initial resistivity, *M* and *N* are two constants related to the intrinsic parameters of the CB itself, and *T* is the temperature.

In the case of temperature or mechanical stimulus, the gap between conductive particles could be expressed separately. When the temperature was relatively lower, the volume expansion of CB could be considered as zero, and the distance between CB was mainly caused by the expansion of PLA matrix. The gap between the CB induced by the temperature *ω_T_*(*t*) could be expressed as *ω*_*T*_(*t*) = *ω*_*T*0_ + *δτt*, where *δ* is the expansion coefficient of the PLA matrix and *τ* is the temperature rising rate. Together, the gap between the CB induced by the mechanical stimuli *ω_F_*(*t*) could be expressed as *ωF*(*t*) = *ρ*_c_ *ht*, where *ρ*_c_ is the curvature of the PISA sample and *h* is the thickness. Then, the resistance change caused by the combination of temperature and mechanical stimulus could be expressed as (Supporting Theory, Supporting Information)

(2) $$\rho \left(t\right)=\rho_{1}\;\exp\left[\frac{M\cdot \left(\omega_{T0}+\delta \tau t\right)+M\cdot \rho_{c}ht}{2\left(\tau t+N\right)}\right]$$

Equation (2) could qualitatively reflect the variation law of PISA sample resistance under the thermal and mechanical stimuli. At the lower temperature, the PISA sample increased with the temperature increasing. The rate of resistance rise was proportional to the rate of temperature rise *τ*↑. When the temperature gradually rose to a certain threshold, the resistance of the PISA sample decreased with the temperature increasing. For mechanical stimulation, the greater the PISA sample curvature *ρ*_c_↑ caused by mechanical stimulus, the greater the resistance *ρ*(*t*)↑, and the resistance was positively correlated with the sample curvature. Mechanical stimulation was performed in a short period of time to form jump points on the resistance curve, which could be seen in Figures 2b,c and 3d–f.

The performances of the above tests were to take the PISA samples as the sensor to passively receive the external signal and reflect the external mechanical signal or temperature signal or bifunctional mechanical‐temperature signal through the resistance. Then the active touching performance as a sensor–actuator was tested. The sensor–actuator performance was tested by another home‐made system. At room temperature, the PISA was rigid and had a wax‐like surface, while above the glass transition temperature (*T*_g_), the structure was pliant and elastomeric. In this state, any deformation applied to the structure could be fixated by cooling below the *T*_g_. Reheating the structure led to a recovery of the original printed shape. The *T*_g_ of the CB/PLA was 61.9 °C (Figure S15, Supporting Information) and the shape memory program was triggered by placing the structure in a medical paraffin oil bath at 100 °C (**Figure** **4**a and Movie S2, Supporting Information). The sample was heated to 90 °C through a heat gun and curved into an arc as the temporary shape. The resistance of the PISA sample was measured with the multimeter. The sample before being curved was tested as the control group experiment first; the details of the testing process are presented in the Experimental Section. The resistance–time curve of the curved and straight PISA had the same rising tendency (Figure S16, Supporting Information). These two resistance–time curves were fitted with a single exponential decay equation. The exponential decay coefficient of the curved PISA was smaller than the straight PISA. In other words, the resistance rising rate of curved PISA was faster than that of straight PISA. The final resistance of the curved PISA was lower than the straight PISA. The main reason was that the sample could not return to its original shape completely (Figure S17, Supporting Information).

**Figure 4:**
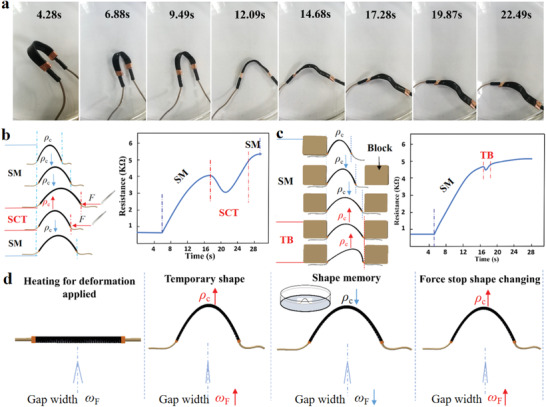
Active touching performance of the PISA in the paraffin oil bath. a) The PISA sample memorized its original shape triggered by the hot paraffin oil bath. b) The curved PISA memorized its original shape and stretched in the hot paraffin oil bath. At this point, tweezers were used to make it bend again. During the bending process, the resistance of the PISA decreased first and then increased. In this process, the curvature *ρ*_c_ of the PISA decreased first and then increased. SM: Shape memory; SCT: shape changing by tweezers. c) Placing a weight block on the shape memory path of the curved PISA, when the PISA shape changed and touched the block, a similar resistance curve was formed, which was first dropped and then raised. The curvature *ρ*_c_ of the PISA also decreased first and then increased. TB: Touching block. d) The illustration reflected the curvature *ρ*_c_ changing of the PISA. The PISA memorized the straight shape and gradually stretched out; the curvature decreased in this process. When it encountered an object, it was subject to the touching force, and the middle part continued to shape changing, increasing the curvature. The gap width (mechanical stain *ω*_F_) or spacing between the CB nanoparticles induced by mechanical stimulus also varied with the curvature.

When the sample was just put into a hot paraffin oil bath, the resistance of the PISA grew exponentially with time due to the simultaneous occurrence of temperature expansion *ω_T_*(*t*)↑ and mechanical stain *ω_F_*(*t*)↑. It was difficult to distinguish whether the increase of resistance was due to mechanical deformation or temperature. In addition, due to the small change of resistance caused by mechanical strain, it was difficult to accurately judge the size of the shape variable, but could roughly judge the change of deformation through the exponential curve growth coefficient. When sufficient time elapsed, the PISA kept the same temperature as the hot paraffin oil bath. It could be assumed that temperature changes had little effect on the resistance of the PISA. At this time, the main factors affecting the resistance of the PISA were the bending and stretching of the device. As shown in Figure 4b, the PISA memorized its original shape stretching forward and the resistance increased gradually. When the PISA sample was bent in the oil bath with tweezers, the resistance–time curve showed a drop in area. At this area, *ρ*_c_↑, the resistance of the PISA decreased. After this period, the resistance of the PISA kept increasing. Similarly, a curve could be obtained by placing a weight block on the path of the deformation in the shape memory of the PISA sample (Figure 4c). This could create the active touching effect in the paraffin oil bath. During the touching process, the block prevented the PISA from stretching forward when the PISA touched the block, but the middle part of the PISA kept changing shape (Figure 4d). Thus, the curvature *ρ*_c_ of the PISA decreased first and then increased, the corresponding resistance of the PISA also increased first and then decreased. The resistance drop area was much shorter, indicating that the curvature change of the sample shape memory was much smaller.

Next, the PISA sample was used to simulate the active touch of a finger to demonstrate the application potentials described in this Communication in humanoid robots (**Figure** **5**a). The PISA sample was curved as a touching style. The temporary shape was curved like a raised finger. Then, the PISA sample changed the shape from curved to straight under a heat gun with a set temperature of 90 °C. The head of the PISA sample would touch the mobile phone screen during the shape changing process. The movement of the PISA sample and the resistance were recorded during the whole touching process (Figure 5b and Movie S3, Supporting Information). From this resistance curve, the different stages in the process of “touching” had been presented. The first stage was the heating and shape change; the resistance of the PISA sample increased in an approximate straight line from 5.88 to 14.37 s. At this stage, both stretching and thermal action increased the resistance of the device, *ω_T_* + *ω_F_* > 0. The second stage was the touching, from 14.37 to 15.77 s; the resistance of the bionic finger had dropped jump (Figure 5c). At this stage, the PISA bending, *ρ*_c_↑, *ω_T_* − *ω_F_* < 0; the resistance of the PISA decreased. After this period, the *ρ*_c_↑, *ω_T_* − *ω_F_* > 0; the resistance of the PISA kept increasing. The third stage was the cooling, from 15.77 s to the end; the resistance of the bionic finger decreased in an approximate straight slope line due to the temperature decreasing rapidly. When the head of the PISA sample touched the screen, it was possible to see the ripple on the mobile phone screen, just like the human finger touching the screen of the mobile phone (Supporting Note and Movie S3, Supporting Information). Because the CB/PLA composite had a good conductivity, the action of PISA could be driven with a current. Temperature could be readily adjusted by changing in amperage, which allowed us to control the morphing speed and final shape of the PISA. By elaborating 4D printed structures and controlling applied currents, we could precisely manipulate the synthetic finger analogs. Single material system combined with 4D printing technology could provide us with broader design space and more possibilities for the application of intelligent devices in the future.

**Figure 5:**
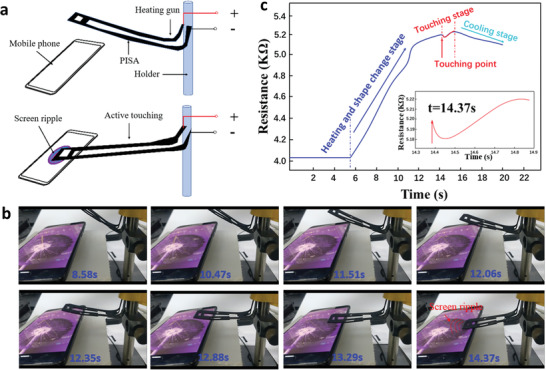
The application of active touching mobile phone screen. a) Schematic of the PISA that mimicked finger of active touching mobile phone and reflected the touching information through changing resistance. b) The PISA was designed to mimic the ability of finger that could actively touch the screen. The PISA actively touched the phone screen and triggered the screen saver creating a ripple (Movie S3, Supporting Information). c) The active touching caused the resistance to decrease first and then increase. The touching point was about 14.37 s at frequency of 4 Hz and at displacement of 8 µm.

In summary, a printed integrated sensor–actuator with bioinspired gaps had been demonstrated with bifunctional sensing ability and active touching ability as triggered by thermal stimulation. First, the materials prepared in this Communication had good electrical conductivity and printability. The gradient gaps from the bottom to the top layer endowed the PISA with mechanical sensing property. Due to the change of the gap between the CB nanoparticles caused by the temperature expansion of the PLA matrix, the PISA was endowed with the property of temperature sensing. The electrical resistance changed by mechanical or temperature action was mainly due to the electron tunneling effect owing to the gap varying between CB nanoparticles. The innovative ability of the PISA was the ability to touch the object actively and get the resistance signal feedback, thus forming the active perception ability. The PISA memorized the original shape and touched the screen of a phone, where the touching force was feedback through the resistance change. Although active touch has achieved certain effects in this research, the feedback mechanical signal is still very small and the sensitivity is low, and it can distinguish mechanical and thermal signals in some situations. This work paves a new prospect for multifunctional sensor and promotes the fusion of future sensor–actuator facilitating application in a variety of intelligent scenarios. Future research will focus on improving the diversity and sensitivity of the sensor–actuator, the differentiation of multiple signal sensations, double‐way actuators, and so on.

## Experimental Section

##### Materials Preparation and Printing Parameters

CB/PLA composite filaments were synthesized by mixed extrusion. First, the composite filaments were composed of 90 wt% pure PLA powder and 10 wt% CB nanoparticles. The CB (BLACK PEARLS 2000, particle size 18 nm, Cabot Co.) and PLA (NatureWorks Co. LLC, USA) powders were dried in a vacuum‐drying oven (Shanghai Pein Experimental Instrument Co., Ltd., China) at 65 °C for 6 h. Subsequently, the dried materials were mixed using a ball mill (Wujin Changxing Machinery Co., Ltd., China). The ball milling process was conducted for 4 h at room temperature to obtain raw materials for extrusion. A double screw extruder (KTE‐20, Nanjing Kerke Extrusion Equipment Co., Ltd., China) was used to produce the FDM filaments. The extrusion process parameters were optimized to ensure that the filament had the same diameter, density, and ratio. The printing filament was set to have a diameter of 1.75 mm and a screw speed of 35 rpm with a constant die temperature and screw rotation speed. All the PISA samples were printed by an FDM (Ultimaker 2, Datacard). Brass nozzles with 0.3 mm diameter were used. The printing speed was 30 mm s^−1^. The temperature of the print nozzle was set to 220 °C. Linear filling method was adopted and the filling rate was 100%. The substrate preheating temperature was 65 °C. To ensure high resolution and good interface adhesion, a paper tape was used to bond to the building plate.

##### Materials Characterization

The morphology of the CB nanoparticles and their distributing in the PLA matrix were characterized by Field Emission Scanning Electron Microscopy (FESEM) (JEOL JSM‐6700F). Energy dispersive spectrum analysis (EDS) was performed using a field emission SEM equipped with an EDS detector (X‐MaxN 150, Oxford, Britain). The sections of the one‐layer, two‐layer, and multilayer samples were obtained by a super deep scene 3D microscope (VHX‐5000, Keyence, Japan).

##### Performance Test as a Mechanical Sensor

The PISA sample was put on the two positioning holders. A rod was pressed down on the middle of the center controlled by a precision electric displacement table (Paidiwei, PP110‐30) with 10 µm positioning accuracy. Frequency response performance of mechanical stimulus test was carried out with a home‐made test system illustrated in Figure S10 (Supporting Information). The vibration generator at frequencies of 0.1, 0.2, 0.5, 1, 2, 5, 10, 20, 50, 100, 200, and 400 Hz was controlled with a digital signal generator (AFG‐2005, GW INSTEK). The displacement of the sensor was simultaneously recorded with a high‐speed video camera (Dimax HD, PCO, Germany) for vision‐based kinematic recognition by software (TEMA MOTION). The resistance of the PISA sample was measured with a multimeter (34465A 612 digit, Agilent), with BenchVue Software for recording and visualization in the whole experiments.

##### Performance Test as a Temperature Sensor

The temperature sensing performance was tested with a constant temperature heating table (XINHAOMAI, Digital Technology) and an infrared thermal imager (Testo 868, Germany). The room temperature PISA sample was put on the heating table with preset maximum temperatures of 60, 80, 100, 120, 150, and 200 °C, respectively. When the sample was heated to its maximum temperature and the resistance no longer changed, it was removed from the heating table and placed in the air to cool naturally. The resistance of the PISA sample was measured with the multimeter in the whole experiment process.

##### Performance Test as a Bifunctional Sensor

The room temperature PISA sample was put on a 50 °C heating table, and then touched the PISA sample with a pen. The resistance of the PISA sample was measured with the multimeter. The temperature of the PISA sample was tested by an infrared thermal imager (Testo 868, Germany). The resistance of PISA was recorded in the whole experiment process.

##### Performance Test as a Sensor–Actuator

The 300 mL medical paraffin oil was put on a large caliber petri dish and heated to 90 °C on a constant temperature heating table. The room temperature straight PISA sample was put into the medical paraffin oil bath first, and the resistance–time curve was recorded simultaneously. Then, the sample was taken out from the paraffin oil bath and cooled to room temperature. Next, the PISA sample was heated to 90 °C through a heat gun (STEINEL, HG‐2310LCD) and curved into an arc as the preset shape. Finally, the curved PISA sample was put into the medical paraffin oil bath, and the resistance–time curve was recorded as the shape recovery process simultaneously. In addition, tweezers and weight blocks were used to stop the PISA stretching in the shape memory process in another experiment cycle. One head part of the PISA sample was attached on a weight block. Then, the whole PISA with weight block was put into the medical paraffin oil bath. In the shape changing process, the tweezers were used to bend the PISA.

##### Active Touching Test

The desktop of a mobile phone (Xiaomi, Note 3) was set as a dynamic desktop, which could produce water ripple effect when touched. The PISA sample was heated to 90 °C through a heat gun and curved to an arc as the preset shape. The PISA sample was fixed on a bracket and the mobile phone was placed under the PISA sample. The PISA sample was connected with the multimeter for electricity supply and resistance recording simultaneously. The heat gun with preset of 90 °C was used to heat the PISA sample to trigger shape recovery for active touching.

## Conflict of Interest

The authors declare no conflict of interest.

## Supporting information

Supporting Information

Supporting Movie 1

Supporting Movie 2

Supporting Movie 3
