# Supplementary material for: 4D Printing Strain Self‐Sensing and Temperature Self‐Sensing Integrated Sensor–Actuator with Bioinspired Gradient Gaps
Source: Adv Sci (Weinh). 2020 May 13;7(13):2000584. doi: 10.1002/advs.202000584 (PMC7341108; doi:10.1002/advs.202000584)
Supplement: Supplementary file 1 — Supporting Information [file ADVS-7-2000584-s001.pdf]

Supporting Information

4D printing strain self-sensing and temperature self-sensing integrated-sensor-actuator with bio-inspired gradient gaps

Daobing Chen, Qingping Liu, Zhiwu Han, Junqiu Zhang, HongLie Song, Kejun Wang, Zhengyi Song, Shifeng Wen, Yan Zhou*, Chunze Yan, Yusheng Shi*

Table S1 Comparison of recent works relating to self-sensing actuator or sensor or actuator

Reference	Materials	Type	Manufacturing method	Feedback signal	Drive mode	Gauge factor	Material Integrated type
[1]	Graphite microparticles and carbon nanotubes (CNTs), Paper, PP film	Self-sensing actuator	Ink Printing, Coating	Resistance	Light, Electrothermal	~45.88	Two layers, separation
[2]	Graphene, Cu foil, Nafion	Self-sensing actuator	Casting	Potential	Current	——	Sandwich structure, separation
[3]	Silvercoated carbon nanofibers, PLA	Sensor or Actuator	4D printing	Resistance	Electrothermal	——	Integration
[4]	Polycaprolactone, organic materials	Sensor or Actuator	Casting	Voltage	Thermal	——	Sandwich structure, separation
[5]	Pt film, polyurethane acrylate (PUA)	Sensor	Depositing	Resistance	——	>2000	Two layers, separation
[6]	Carbon black, paper	Sensor	Ink Printing, Coating	Resistance	——	~647	Two layers, separation
[7]	PDMS film, Au	Sensor	Depositing	Resistance	——	~5888	Two layers, separation
[8]	Ag/silicone inks	Sensor	3D printing	Current	——	——	Integration
[9]	Silicone, ionic conductive ink	Sensor	3D printing	Capacitive	——	——	Multilayers, separation
[10]	Sylgard 184 PDMS	Actuator	Soft lithography moulding and 3D printing	——	Gas	——	Multilayers, separation
[11]	Hydrogel	Actuator	4D printing	——	Water	——	Integration
[12]	TPU, ABS	Actuator	4D printing	——	Force	——	Two layers, separation
[13]	Liquid Crystal Polymer	Actuator	Casting	——	Electrothermal	——	Sandwich structure, separation
[14]	SMP, Fe3O4, NdFeB	Actuator	Commixture	——	Magnetic	——	Integration

[15]	AgNWs, paper, polypropylene film	Actuator	4D printing	____	Light, humanity, electrothermal	____	Two layers, separation
[16]	SMP, Oligo, W18O49 nanowires	Actuator	synthesis	____	Photothermal	____	Integration
This work	PLA, Carbon Black	Self-sensing actuator	4D printing	Resistance	Thermal	~48.46	Integration

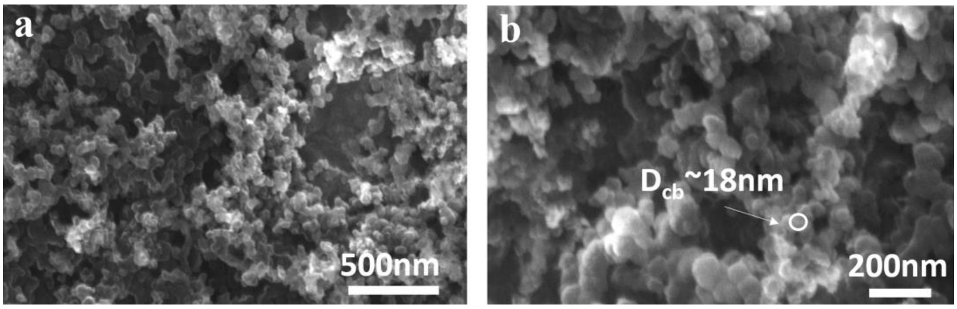

Figure S1. Carbon black nano particles. a)-b) The FESEM image of the CB nano particles shows that the diameter of a CB nano particle is about 18nm.

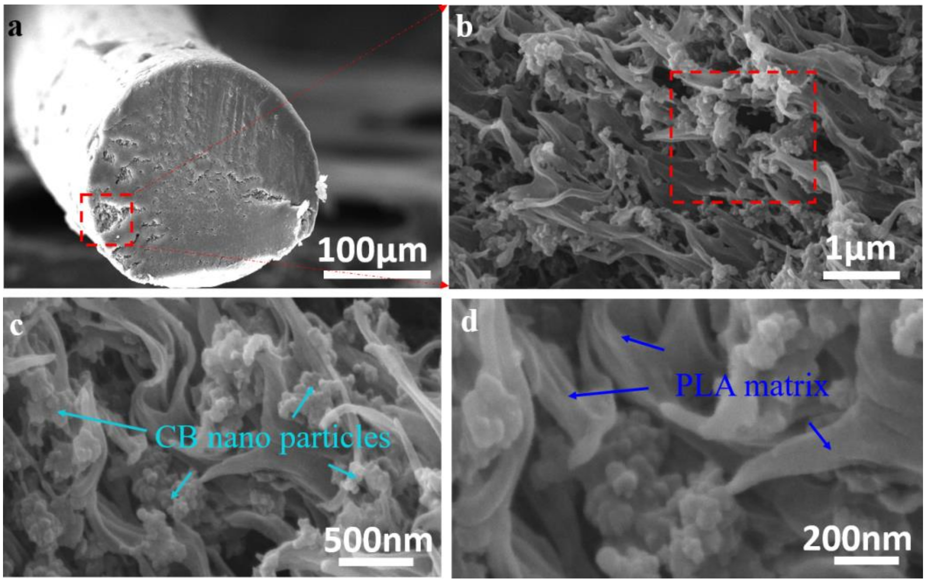

Figure S2. The distribution of carbon black nano particles in PLA matrix. a) It can be seen from the cross section of the CB/PLA composite material, carbon black nanoparticles are well dispersed in the PLA matrix. b) PLA appears filamentous, and carbon black nanoparticles are distributed among

these filaments. c)-d) Most carbon black nanoparticles combine into clumps or chunks. These clumps or chunks range in size from 200nm to 500 nm.

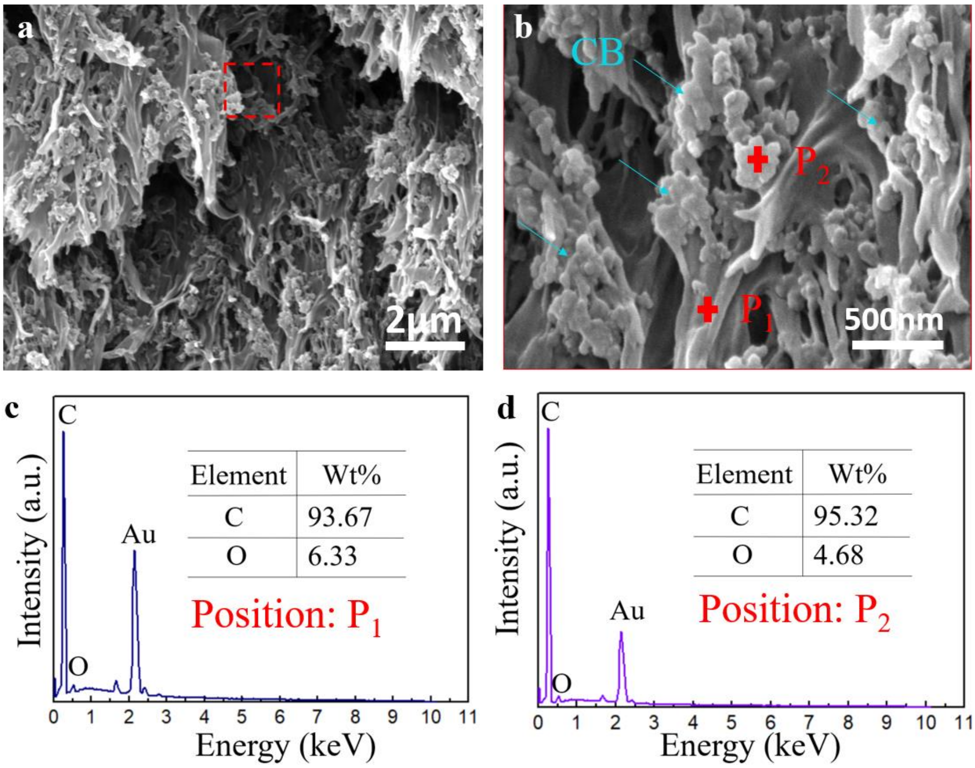

Figure S3. The Energy Dispersive Spectrum Analysis (EDS) of the CB/PLA composite materials. Since PLA contains oxygen element, it can be distinguished from carbon black by the content of oxygen element. a)-b) In test position 1 and position 2, the oxygen content was 6.33% (c) and 4.68% (d), respectively. It indicated that the bulk or granular is the CB nanoparticles and the filamentous is the PLA.

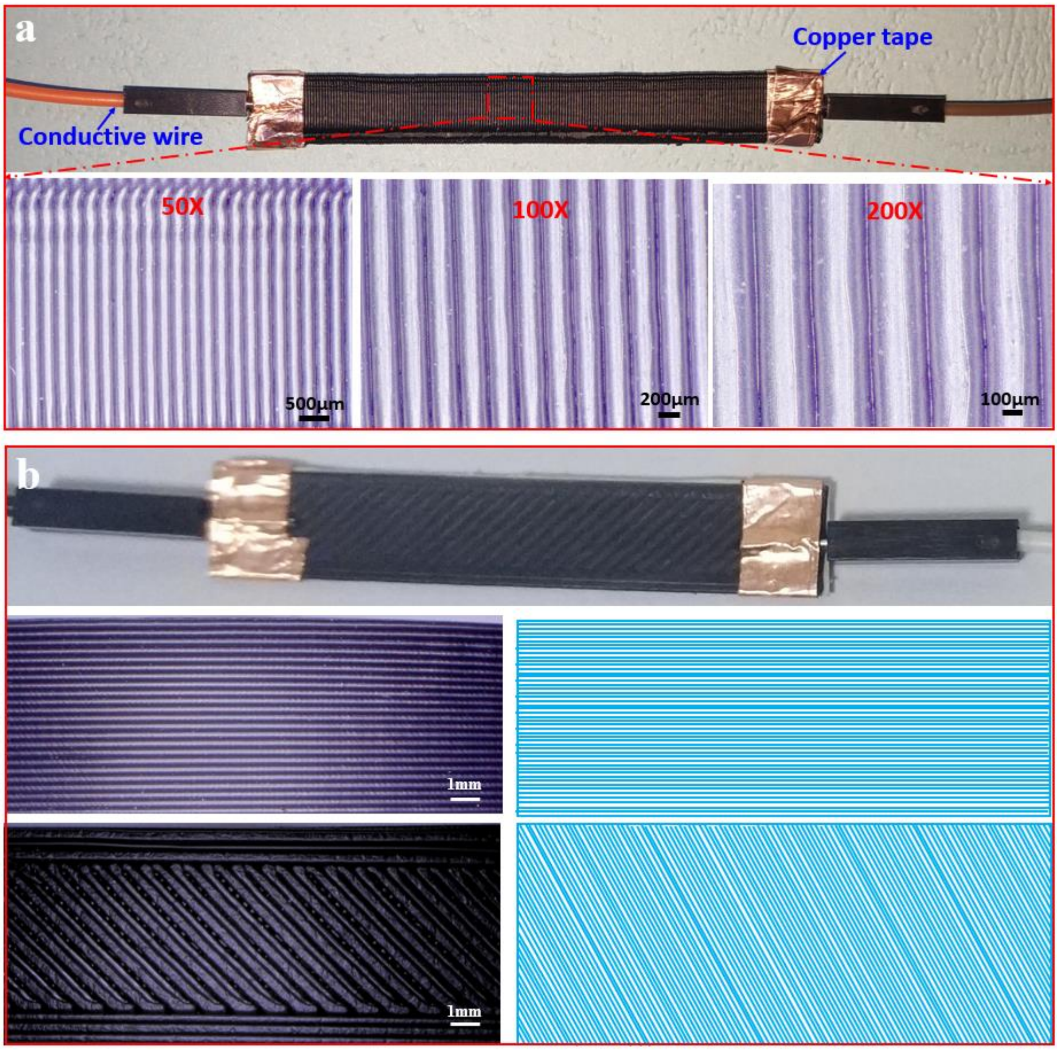

Figure S4. Optical microscope images of the PISA samples. The filaments on the PISA samples arranged in parallel to form many arranged gaps. a) Linear filling PISA sample with 0°. b) Linear filling PISA sample with 90° and 45°.

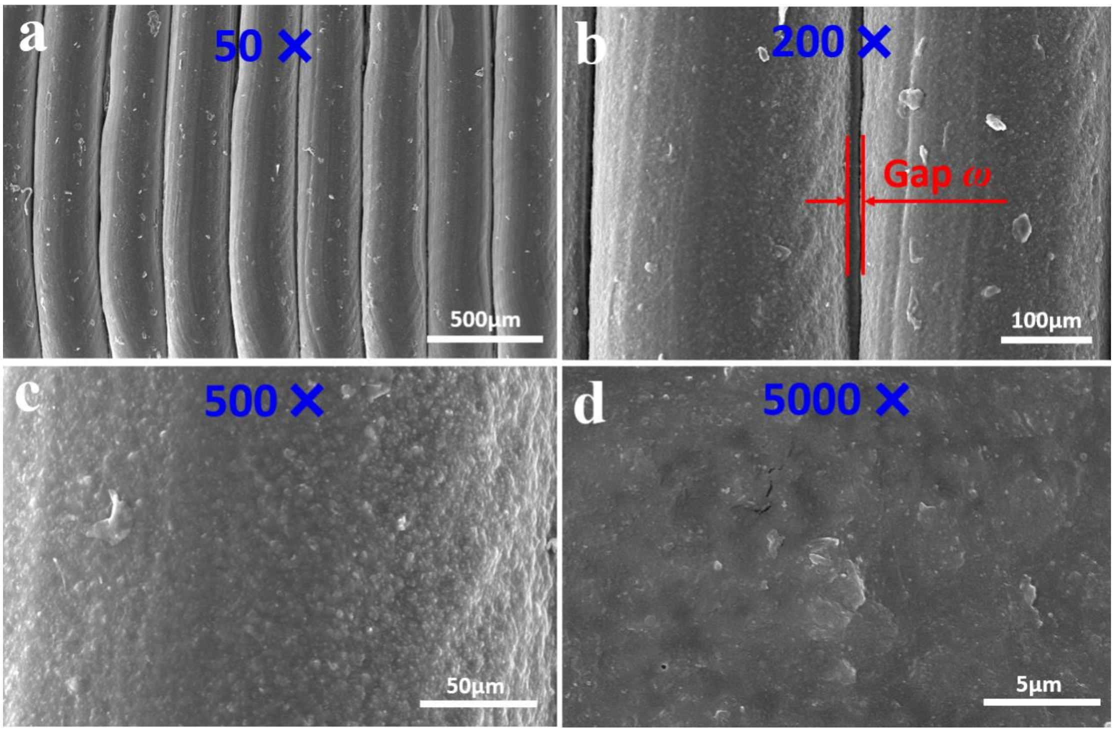

Figure S5. SEM images of the filaments on the PISA sample. a) The round line strands extrusion from the FDM arranged and formed the three-dimensional entity. b) Gaps formed in the arrangement of the strands, inevitably. The width of the gap was about ~10μm. c)- d) Carbon black nano particles were embedded in the filaments.

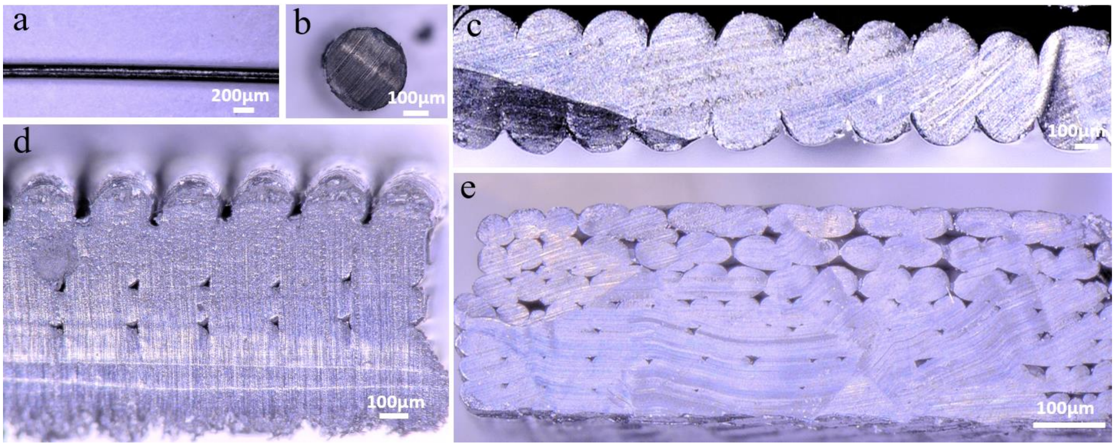

Figure S6. The PISA sample was fabricated by FDM with linearly filling method. a) the single strand of the CB/PLA. b) the section of a single strand. c) the single layer. d)-e), In a PISA sample of

four or multilayers, gap structures were formed between the filaments of the upper layers. The gap width had the characteristic of increasing gradient from bottom layer to top layer.

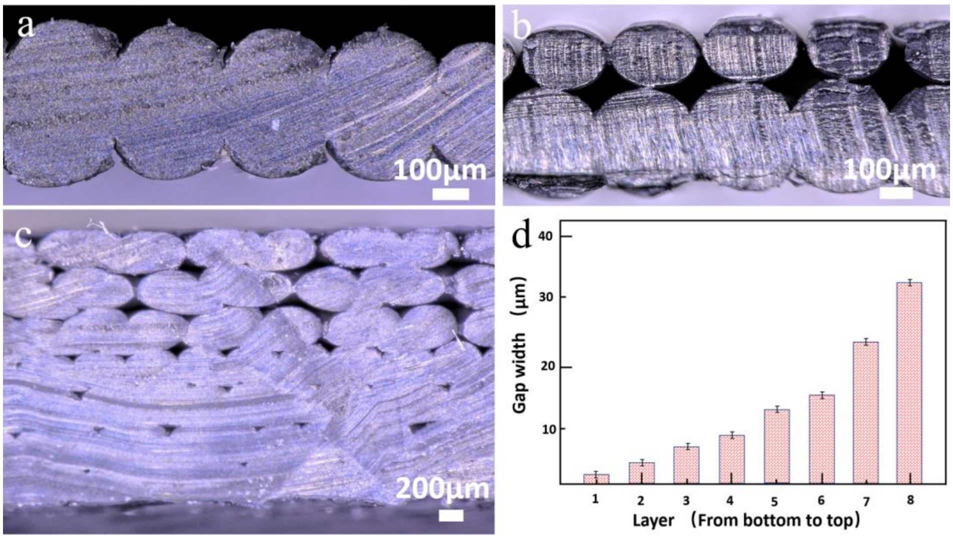

Figure S7. Optical microscope images of the section of PISA samples with different layers. a) One layer. b) Double layer. c) Multi-layer. d) The maximum gap width of the filaments from the bottom layer to top layer. From the point of view of the extruded monofilament, the single CB/PLA filament was smooth and round with good printability. The diameter of a single extruded CB/PLA filament was about 250 μm. Filaments in single layer closely connected with each other, the round filaments were extruded into an elliptical structure and large chord length contact was formed. In the second layer of the double layer sample, the diameter filament was smaller than the first layer and arranged as sparse pattern, there were a lot of gaps between filaments. In the multilayer sample, the rule can be exhibited more intuitive, the gap between filaments become larger and larger from the bottom layer to the top layer. This is because the preheating heat of the substrate and the residual heat of the upper layer produced a thermal impact on the next layer, making the next layer in a soft state. Under the effect of gravity and the thermal pressure of the upper layer, the filament deformed and merged with the two adjacent filaments in the same layer, making the gap between the filaments in this layer reduced.

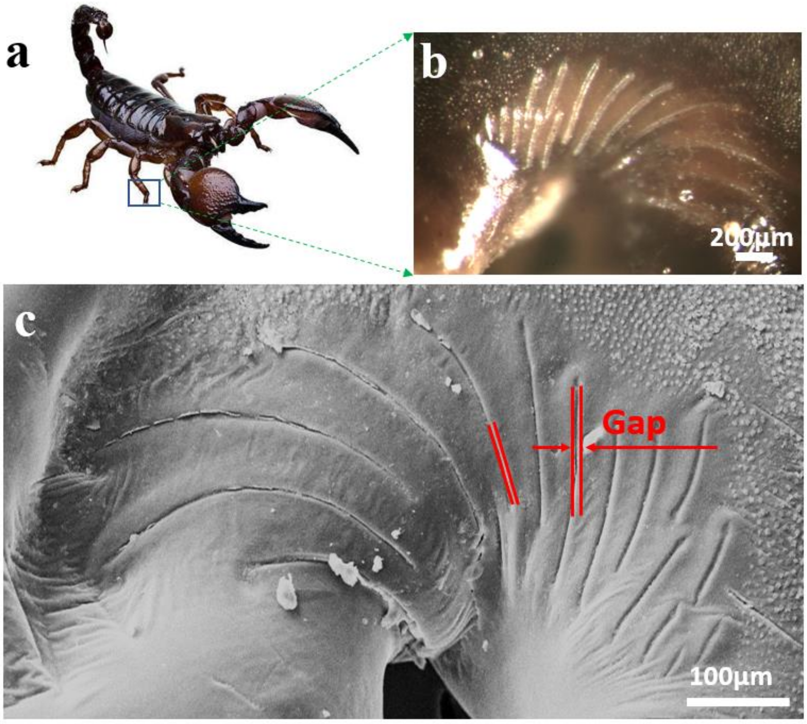

Figure S8. Lyriform organ on scorpion (*Heterometrus petersii*) legs [17]**.** a) Scorpions can detect tiny vibration generated by prey or predators through the lyriform organ embedded into the exoskeleton of the legs. b)- c) The lyriform organ is formed of gaps with different length and structure. It contains the straight gap and the curved gap. The width of the gap is about 2μm. The lyriform organ responds to mechanical disturbance by converting vibration stimulus into deformation of the lyriform organ. The deformation of the lyriform gap transfers the mechanical signal into the bioelectric signal. The gap structure can amplify the mechanical signal through the stress concentration effect [17]. (Scorpions were purchased from pet stores.The scorpion was cleaned by ultrasonic wave after death by diethyl ether. After drying, the lyriform sensors were observed by a super deep scene 3D microscope (VHX-5000, Keyence, Japan) and filed emission scanning electron microscope (FESEM, JEOL JSM-6700F), respectively.)

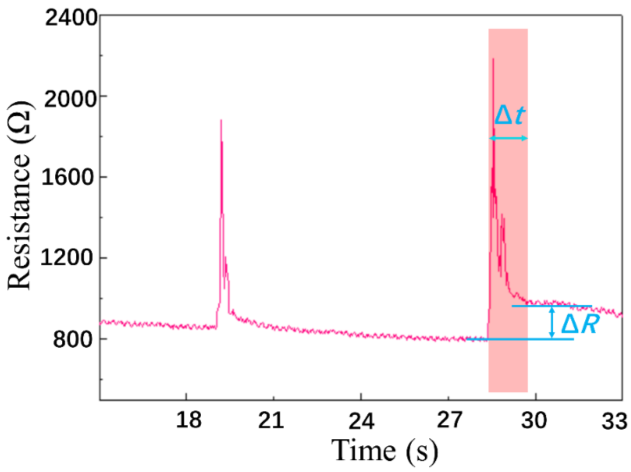

Figure S9. The finger touching test. The PISA sample can reflect the touching information about the finger. The signal jump height (the change in resistance) can reflect the touching force. The time of one jump (Δt) can reflect the contact time. The stationary resistance difference (ΔR) before and after contact can reflect the temperature rise.

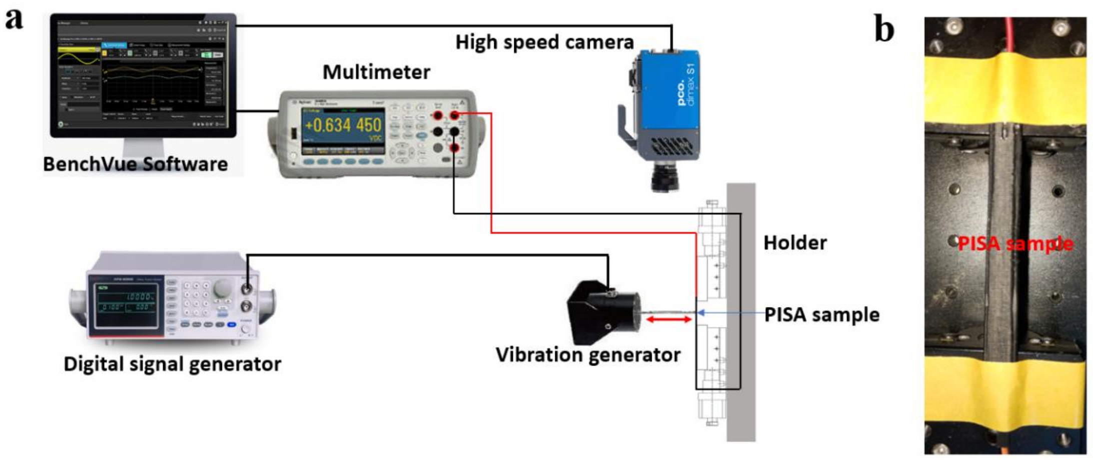

Figure S10. The experimental set-up for obtaining the response signal (electrical signal and displacement signal) of PISA samples. a) Schematic illustration of the mechanical stimulus frequency response performance test by the home-made system. [6, 7] It mainly contains the digital signal generator, vibration generator, multimeter, high speed camera, computer with BenchVue Software and holders. The vibration generator is controlled by the digital signal generator to produce vibration signals of a certain frequency and waveform. The rod on the vibration generator vibrates

back and forth, transmitting the mechanical vibration signal to the PISA sample. The resistance change of the PISA sample was measured with a multimeter, with BenchVue Software for recording and visualization in the whole experiments. The displacement of the sensor was simultaneously recorded by a high-speed video camera and motion analysis software for vision-based kinematic recognition.[2] b) A PISA sample was ready for tests.

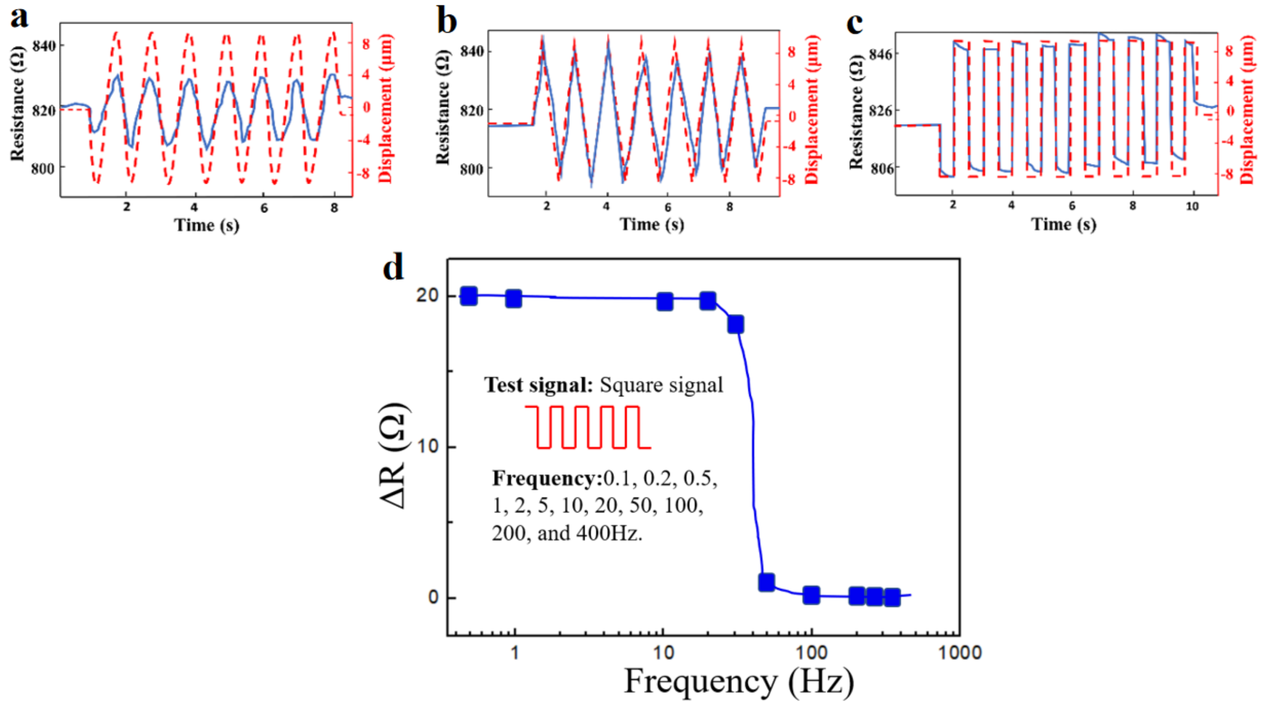

Figure S11. The mechanical stimulus signal frequency response of the PISA. a)-c) The PISA can relatively accurately reflect different waveform signals. a) Sine wave. (b) Triangle wave. c) square wave. d)In the case of low frequency, less than 20 Hz, the PISA sample can well reflect the mechanical signal applied. When the frequency increases, the resistance of the sample basically remains unchanged, unable to reflect the change of the mechanical signal. The main reason was that high frequency mechanical signals would generate more heat, and the heat can not spread out in time. Thus, the mechanical signals and thermal signals were superimposed on each other, unable to

distinguish the signals. At this point, the PISA sample was only suitable for measuring mechanical signals at low frequencies.

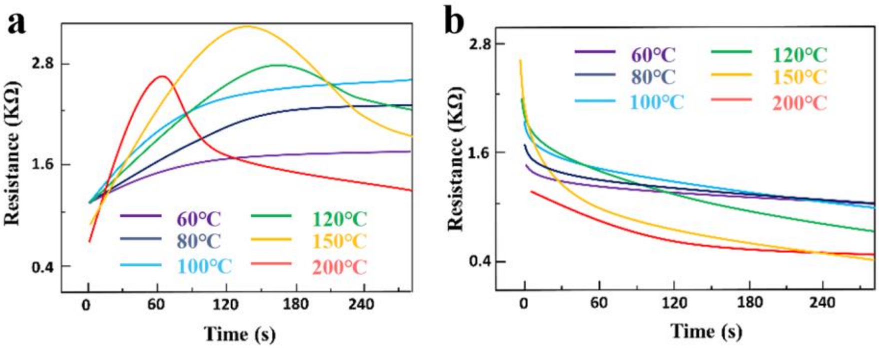

Figure S12. a) The resistance-time curves of the PISA heating with different maximum temperature (Max. *T*), 60, 80, 100, 120, 150, 200℃. b) The resistance-time curves of the PISA cooling from different Max. *T*.

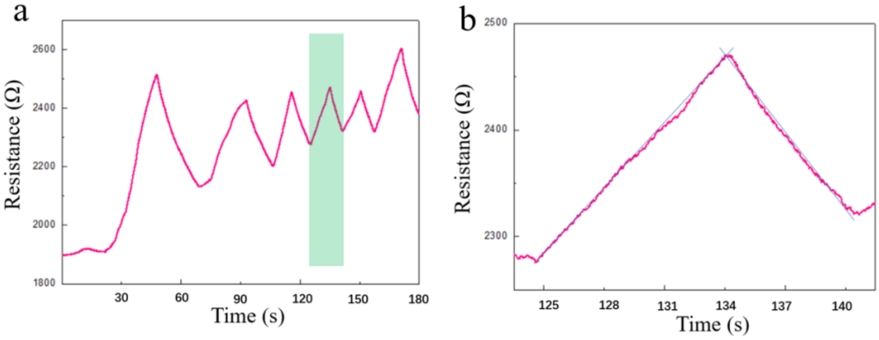

Figure S13. a) Resistance-time curve of the PISA obtained by the approach and withdrawal of heat sources. b) In a short period of time, the resistance raised in a straight line.

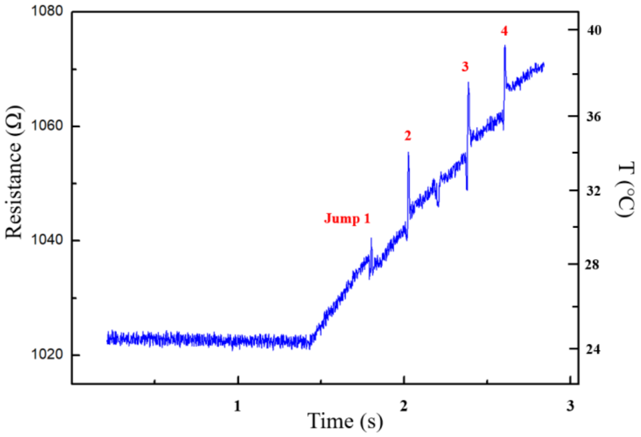

Figure S14. The PISA sample can reflect the mechanical stimulus while the temperature changes. The PISA resistance increasing of the jump was induced by the mechanical stimulus from the top to the bottom side.

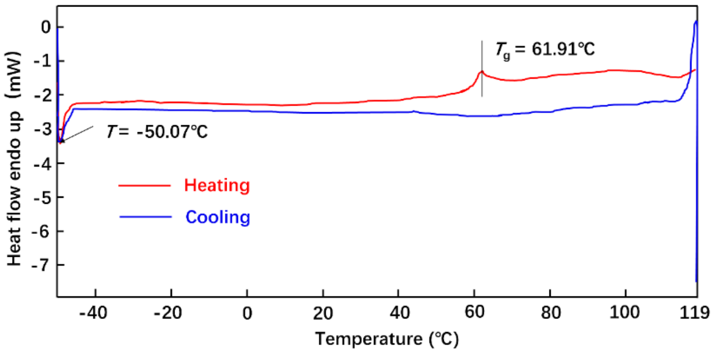

Figure S15. Differential scanning calorimetry (DSC) analysis. Differential scanning calorimeter (DSC) measurements were conducted using a TA DSC-Q100 to examine the glass transition temperature (T_g) of the CB/PLA composites. The samples were tested from -50 °C to 119 °C under a nitrogen atmosphere at the same heating and cooling rates of 5 °C min^{-1} in two scans. The samples showed a thermal transition near −50.07 °C and an obvious endothermal peak at 61.91 °C in the heating run due to the glass transition of elastomer and melting of PLA, respectively.

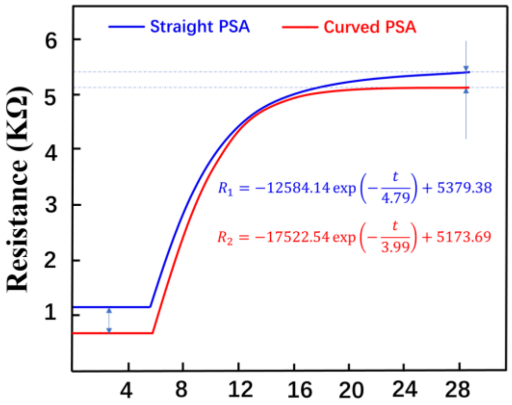

Figure S16. The resistance-time curve of the curved PISA and the straight PISA had the same rising tendency. The differences are on the initial and final resistance and growth rate. The initial resistance of the curved PISA was about 700 Ω, which was lower than the straight PISA, it was about 1100 Ω. These two resistance-time curves were fitted with a single exponential decay equation. The exponential decay coefficient of the curved PISA is smaller than the straight PISA, -1/3.99< -1/4.79. In other words, the resistance growth rate of curved PISA was faster than that of straight PISA. The final resistance of the curved PISA was lower than the straight PISA, the main reason was that the sample did not return to its original shape 100%.

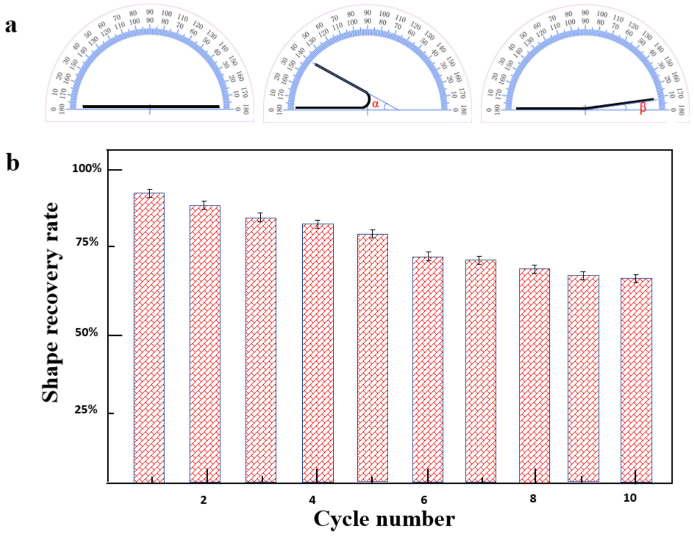

Figure S17. The shape recovery rate tests of CB/PLA composite. a) The test method and shape recovery rate calculation method. In the test, the shape recovery rate (R_r) is calculated by [3,18]:

$$R_r = \frac{180 - \alpha - \beta}{180 - \alpha} \times 100\% \qquad (S1)$$

b) The shape recovery rate of CB/PLA composite in different cycle number.

Supporting theory

According to Sheng P's formula [19, 20] based on the theory of electronic tunneling effect, the conductivity σ of the CB/PLA composite can be expressed as:

$$\sigma = \sigma_0 exp\left(\frac{-E_1}{T + E_0}\right) \tag{S2}$$

where E_1 is the energy required for an electron to cross the gap between conductive filler particles, $E_1 = \omega A\varepsilon_0^2/8\pi k$, E_0 determines the resistance to temperature at very low temperatures, $E_0 = 2E_1/\pi\gamma\omega$, A is the clearance area between CB particles, $\varepsilon_0 = 4V_0/em$, k is Boltzmann constant, ω is the gap between the CB particles, $\gamma = \sqrt{4\pi m V_0/h^2}$, m is the mass of the electron, h is the Plank constant, V_0 is the potential barrier, e is the electric quantity of an electron. At room temperature, since E_0 is far less than T, E_0 and E_1 can be ignored. Therefore, equation (S2) can be approximately expressed as:

$$\sigma = \sigma_0 exp\left(-\frac{1}{T}\right) \tag{S3}$$

It can be seen from equation (S3) that the conductivity of conductive polymer composites increases with the increase of the ambient temperature, that is, the resistivity is inversely proportional to the ambient temperature, and the reciprocal of the temperature is functionally related to the ambient temperature.

Since equation (S3) only has two variables T and ω, let $M = A\varepsilon_0^2/8\pi k$, $N = A\varepsilon_0^2/4\pi^2\gamma k$, the equation (S1) can be changed as:

$$\sigma = \sigma_0 exp\left(\frac{-M\omega}{T + N}\right) \tag{S4}$$

The conductivity σ of the CB/PLA composite is inversely proportional with resistivity ρ, then the resistivity ρ of the CB/PLA composite can be expressed as:

$$\rho = \rho_0 exp\left(\frac{M\omega}{T + N}\right) \tag{S5}$$

It can be seen from equation (S5) that the resistivity of conductive CB/PLA composite is related to the temperature T and the gap ω between conductive CB particles. The resistivity ρ decreases with the

increase of temperature T in inverse proportion, and increases with the increase of the gap ω between conductive filler particles in direct proportion. In the actual process, the change of temperature $T(t)$ and the change of conducting CB particle gap $\omega(t)$ is a function of time, so the resistivity $\rho(t)$ can be rewritten as:

$$\rho(t) = \rho_0 exp\left(\frac{M\omega(t)}{T(t)+N}\right) \tag{S6}$$

The gap between carbon black nanoparticles is caused by a combination of two factors: the first is the different expansion of the composites under the influence of temperature $\omega_T(t)$, and the second is the strain caused by mechanical stimulus $\omega_F(t)$. The equation (S6) can be changed as:

$$\rho(t) = \rho_0 exp\left\{\frac{M[\omega_T(t)+\omega_F(t)]}{T(t)+N}\right\} \tag{S7}$$

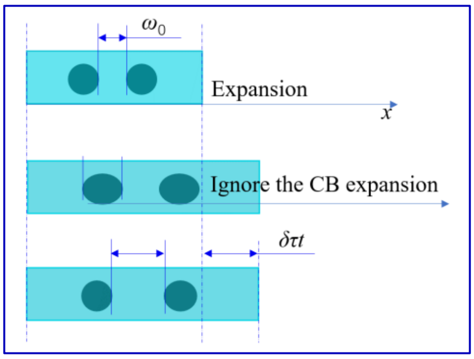

Figure S18. The illustration of the gap between the CB nano particles induced by thermal expansion. As the temperature rises, different materials have different thermal expansion coefficients. At the lower temperature range, the linear coefficient of thermal expansion of CB is less than that of the PLA. When the external temperature rises, the change of electrical resistance of CB/PLA composite material is mainly reflected in the expansion of the PLA matrix material at the lower temperature range (Figure S18). The conducting CB particle gap $\omega_T(t)$ induced by the temperature rising can be expressed as:

$$\omega_T(t) = \omega_{T0} + \delta\tau t \tag{S8}$$

where δ is the expansion coefficient of the PLA matrix, ω_{T0} is the initial gap between the CB nano particle, τ is the temperature rising rate. Then, the equation (S6) can be changed as:

$$\rho(t) = \rho_1 exp\left[\frac{M \cdot (\omega_{T0} + \delta\tau t)}{\tau t + N}\right] \tag{S9}$$

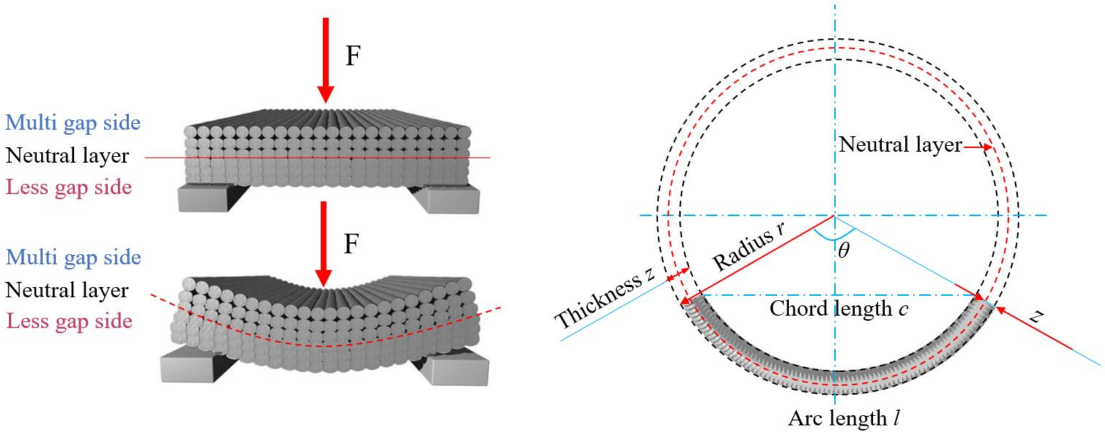

Figure S19. The gap between the CB nano particles induced by mechanical stimulus. Together, the strain ε of the PISA sample induced by the force was estimated. The Figure S19 is the model of the bend PISA when enduring compression strain. The sensor was tailored to be 50 × 8 × 2 mm, with constant arc length l = 50 mm. The PISA sample was bend with radius r, into chord length c, thickness h, central angle θ, and the distance from bottom surface of the sensor to the neutral layer is z. Relationships between θ, r, c and l can be expressed as:

$$\theta \cdot r = l \tag{S10}$$

$$r \cdot \sin\left(\frac{\theta}{2}\right) = \frac{c}{2} \tag{S11}$$

When applying compressive force, the sensor was bend into a certain central angle θ_1 with radius r_1, while the initial was θ_0 and r_0. Therefore, the compression strain ε can be described as follows:

$$\varepsilon = \frac{\Delta l}{l} = \frac{l_1 - l_0}{l_0} = \frac{\theta_1(r_1 - z) - \theta_0(r_0 - z)}{\theta_0(r_0 - z)} \tag{S12}$$

In this process, $\theta_1 \cdot r_1 \approx \theta_0 \cdot r_0$, $r_1 \gg z$ and the initial $r_0 \rightarrow \infty$ when the sensor is flat, the equation (S3) can be simplified as:

$$\varepsilon = \frac{z(\theta_0 - \theta_1)}{\theta_0 r_0} \tag{S13}$$

We suppose that the neutral layer is in the middle of the sensor ($z = h/2$). Subsequently, the compressive strain can be expressed as [6]:

$$\varepsilon = -\frac{h}{2r} \tag{S14}$$

While enduring tensile force, the situation is similar. The tensile strain can be expressed as:

$$\varepsilon = \frac{h}{2r} \tag{S15}$$

The resistivity ρ of the CB/PLA composite induced by mechanical stimulus can be expressed as:

$$\rho(t) = \rho_1 exp\left(\frac{M \cdot \frac{h}{2r} t}{\tau t + N}\right) \tag{S16}$$

Substitution of equation (S8) and equation (S15) into equation (S7) yields:

$$\rho(t) = \rho_1 exp\left[\frac{M \cdot (\omega_{T0} + \delta\tau t) + M \cdot \frac{h}{2r} t}{\tau t + N}\right] \tag{S17}$$

The 1/r is the curvature ρ_c of the PISA sample. Then, the equation (S17) can be rewritten as:

$$\rho(t) = \rho_1 exp\left[\frac{M \cdot (\omega_{T0} + \delta\tau t) + M \cdot \rho_c h t}{2(\tau t + N)}\right] \tag{S18}$$

Supporting note

Capacitive touch screen technology works by using the body's current induction. The capacitive touch screen is a four-layer composite glass screen. The inner surface of the glass screen and the interlayer are coated with nano indium Sikkim oxide (ITO). The outermost layer is a thin layer of silica glass protective layer. When the finger touches the metal layer, the user and the touch screen surface form a coupling capacitance due to the human electric field. For high-frequency current, the capacitance is a direct conductor, so the finger sucks a small current away from the contact point. This current flow from the electrodes at the four corners of the touch screen, and the current flowing through these four electrodes is proportional to the distance from the finger to the four corners. The controller calculates the precise ratio of these four currents to obtain the position of the touch point.

Therefore, to trigger the capacitive screen reaction, several conditions need to be met. The first condition is that the object touching the screen has the characteristic of conducting electricity; the second condition is that the object touching the screen needs high-frequency current to pass through; the third condition is that the object touching the screen needs enough contact surface and contact force.

For PISA, the PISA material itself has good conductivity, the other two conditions depend on the design of the PISA sample. Carbon black particles are embedded in the PLA, and their distribution on the surface largely determines the electrical conductivity of the PISA when it comes into contact with the mobile phone screen. The whole PISA sample was designed as along flat strip. The curved part is close to the fixed position so that the sample of sufficient length is extended outwards. The long-extended part can make the head get larger linear velocity during the deformation process. The rapid temperature rise accelerated the shape memory effect of the sample, further increasing the linear velocity of the head. The head has a hole structure in order to obtain sufficient contact area when the

sample head is in contact with the mobile phone screen. During the active contact shape change process of the PISA, the curvature $\rho_c \downarrow$ of the PISA gradually decreased, so that the sample resistance $\rho(t) \uparrow$ gradually increased. When the sample touches the mobile phone screen, the sample curvature $\rho_c \uparrow$ increased, and the sample resistance $\rho(t) \downarrow$ decreased and then increased (Figure S20).

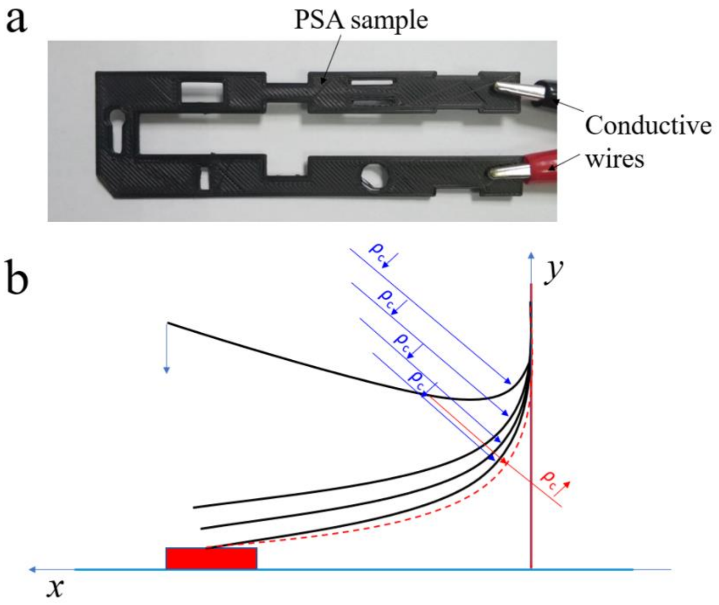

Figure S20. The PISA sample for active touching experiment and illustration of touching process.

a) The PISA sample for active touching experiment. b) Illustration of touching process.

Movies

Movie S1. Sample dynamic touch mobile phone triggered screen feedback. The PSA sample has good electrical conductivity. Since the screen has the effect of touch vibration feedback, when the sample touches the screen, the screen vibration feedback makes the sample repeatedly touch the screen, thus forming the dynamic repeated touch ripple effect.

Movie S2. Shape memory effect of PISA sample in the medical paraffin oil bath. The PISA sample can quickly return to its original shape under the action of hot medical paraffin oil bath. Paraffin oil does not conduct electricity and will not affect the resistance of the PISA samples.

Movie S3. The process of the active touching test of a PISA sample. The curved PISA sample memory its original shape triggered by thermal stimulation. When the head of the PISA sample touches the screen, the mobile desktop creates a ripple effect. At the same time, the touch state can be reflected by the resistance decreasing.

References

[1] M. Amjadi, M, Sitti, *Adv. Sci.* **2018**, *5*, 1800239.

[2] R. Tabassian, V. H. Nguyen, S. Umrao, M. Mahato, J. Kim, M. Porfiri, Il-Kwon Oh, *Adv. Sci.* **2019**, *6*, 19070137.

[3] L. Miao, J. Wan, Y. Song, H. Guo, H. Chen, X. Cheng, H. Zhang, *ACS Appl. Mater. Interfaces* **2019**, *11*, 39219.

[4] R. Liu, X. Kuang, J. Deng, Y. Wang, A. C. Wang, W. Ding,Y. Lai, J. Chen, P. Wang, Z. Lin, H. J. Qi, B. Sun, Z. Wang, *Adv. Mater.* **2018**, *30*, 1705195.

[5] D. Kang, P. V. Pikhitsa, Y. W. Choi, C. Lee, S. S. Shin, L. Piao, B. Park, K. Suh, T. Kim, M. Choi, *Nature* **2014**, *516*, 222.

[6] H. Song, J. Zhang, D. Chen, K. Wang, S. Niu, Z. Han and L. J. N. Ren, *Nanoscale* **2017**, *9*, 1166.

[7] Z. Han, L. Liu, J. Zhang, Q. Han, K. W., H. Song, Z. Wang, Z. Jiao, S. Niu, L. Ren, *Nanoscale* **2018**, *10*, 15178.

[8] S. Guo, K. Qiu, F. Meng, S. H. Park, M. C. McAlpine, *Adv. Mater.* **2017**, *29*, 1701218.

[9] A. Frutiger, J. T. Muth, D. M. Vogt, Y. Mengüç, A. Campo, A. D. Valentine, C. J. Walsh, J. A. Lewis. *Adv. Mater.* **2015**, *27*, 2248.

[10] M. Wehner, R. L. Truby, D. J. Fitzgerald, B. Mosadegh, G. M. Whitesides, J. A. Lewis, R. J. Wood, *Nature* **2016**, *536*, 451.

[11] B. Grigoryan1, S. J. Paulsen1, D. C. Corbett, D. W. Sazer1,C. L. Fortin, A. J. Zaita1, P. T. Greenfield1, N. J. Calafat1, J. P. Gounley, A. H. Ta1, F. Johansson, A. Randles, J. E. Rosenkrantz, J. D. Louis-Rosenberg, P. A. Galie, K. R. Stevens, J. S. Miller1, *Science* **2019**, *364*, 458.

[12] J. A. Faber, A. F. Arrieta, A. R. Studart1, *Science* **2018**, *359*, 1386.

[13] Y. Xiao, Z. Jiang, X. Tong, Y. Zhao, *Adv. Mater.* **2019**, *31*, 1903452.

[14] Q. Ze, X. Kuang, S. Wu, J. Wong, S. M. Montgomery, R. Zhang, J. M. Kovitz, F. Yang, H. J. Qi, R. Zhao, *Adv. Mater.* **2019**, 1906657.

[15] M. Amjadi, M. Sitti, *Acs nano*, **2016**, *10*, 10202.

[16] Y. Zhou, J. Tan, D. Chong, X. Wan, J. Zhang, *Adv. Funct. Mater.* **2019**, *29*, 1901202.

[17] K. Wang, J. Zhang, H. Song, Y. Fang, X. Wang, D. Chen, L. Liu, S. Niu, Z, Yao, Z, Han, L. Ren, *Adv. Funct. Mater.* **2019**, *29*, 1807693.

[18] H. Wei, X. Cauchy, I. O. Navas, Y. Abderrafai, K. Chizari, U. Sundararaj, Y. Liu, J. Leng, D. Therriault, *ACS Appl. Mater. Interfaces* **2019**, *11*, 24523.

[19] P. Sheng, *Phys. Rev. B* **1980**, *21*, 347.

[20] E. K. Sichel, J. I. Gittleman, P. Sheng, *Phys. Rev. B* **1978**, *18*, 5712.
